# Biological Oxidations and Nitrations Promoted by the Hemin–Aβ_16_ Complex

**DOI:** 10.3390/antiox12071319

**Published:** 2023-06-21

**Authors:** Silvia De Caro, Giulia De Soricellis, Simone Dell’Acqua, Enrico Monzani, Stefania Nicolis

**Affiliations:** 1 Department of Chemistry, University of Pavia, Via Taramelli 12, 27100 Pavia, Italy; silvia.decaro01@universitadipavia.it (S.D.C.); giulia.desoricellis@unipv.it (G.D.S.); simone.dellacqua@unipv.it (S.D.); 2 IUSS School for Advanced Studies of Pavia, Piazza della Vittoria 15, 27100 Pavia, Italy

**Keywords:** hemin, β-amyloid, Alzheimer’s disease, oxidative stress, nitrative stress, neurodegeneration, peroxidase

## Abstract

Both β-amyloid (Aβ) peptides and oxidative stress conditions play key roles in Alzheimer’s disease. Hemin contributes to the development of the disease as it possesses redox properties and its level increases in pathological conditions or traumatic brain injuries. The aim of this work was to deepen the investigation of the reactivity of the hemin–Aβ_16_ complex, considering its ability to catalyze oxidation and nitration reactions. We performed kinetic studies in the presence of hydrogen peroxide and nitrite with phenolic and catechol substrates, as well as mass spectrometry studies to investigate the modifications occurring on the peptide itself. The kinetic constants were similar for oxidation and nitration reactions, and their values suggest that the hemin–Aβ_16_ complex binds negatively charged substrates with higher affinity. Mass spectrometry studies showed that tyrosine residue is the endogenous target of nitration. Hemin degradation analysis showed that hemin bleaching is only partly prevented by the coordinated peptide. In conclusion, hemin has rich reactivity, both in oxidation and nitration reactions on aromatic substrates, that could contribute to redox equilibrium in neurons. This reactivity is modulated by the coordination of the Aβ_16_ peptide and is only partly quenched when oxidative and nitrative conditions lead to hemin degradation.

## 1. Introduction

Neurodegenerative diseases are a group of neurological pathologies consisting of neuronal dysfunction and death in the brain and are characterized by a progressive and usually unstoppable clinical course [1,2]. In neurodegeneration, each pathology initially only affects particular neurons so that a selective neuronal vulnerability can be observed; then, the pathologies worsen over time and spread to other areas of the brain in a predictable way [3].

Alzheimer’s disease (AD) is one of the most widespread neurodegenerative pathologies; it represents the most common type of dementia and is characterized by multifactorial etiology [4,5,6,7,8]. AD is associated with the progressive loss of cognitive functions and episodic memory and behavioral and physical disabilities, which can lead to death [4,5]. The brain of a patient affected by AD is characterized by the accumulation of fibrillar peptides (called senile plaques) and neurofibrillary tangles in specific areas, which are the medial temporal lobe and neocortical structures [4,5]. Senile plaques are composed of mainly β-amyloid (Aβ) peptides containing 40–42 amino acid residues, deriving from proteolysis due to the action of β- and γ-secretases of the amyloid precursor protein (APP), a transmembrane protein found in both cell and organelle membranes [4]. Aβ deposition usually starts in neurons which express high levels of the gene which encodes for APP [3]. Senile plaques are extracellular deposits, but Aβ can also accumulate inside the cell [4].

Metal dyshomeostasis plays a crucial role in neurodegenerative diseases. Although several metals, including transition metals such as zinc, copper, and iron, are essential for several physiological functions and for life [2], their homeostasis must be strictly regulated because the accumulation or deficiency of metals can lead to pathological conditions.

High concentrations of zinc, copper, and iron have been found in the brain area affected by AD [4,9]. Dishomeostasis of these metals is implied in Aβ aggregation since the hydrophilic N-terminal region of Aβ contains binding sites for these three metals [9]. Moreover, redox-active metal ions are involved in the onset of oxidative stress that precedes amyloidogenesis [9]. In particular, iron ions catalyze the formation of reactive oxygen species (ROS), such as superoxide, hydrogen peroxide, and above all, hydroxide radicals (OH^•^) that can generate lipid peroxidation and nucleic acid adducts, through Fenton chemistry, which are characteristic of AD [4,9]. For this reason, brain cells have several mechanisms to prevent oxidative stress, such as the presence of glutathione and thioredoxin/peroxiredoxin systems, superoxide dismutase, catalase, and reducing agents such as α-tocopherol and ascorbate [4,9]. However, an imbalance due to high levels of oxidant factors or low levels of antioxidant systems can cause cell death.

Under oxidative stress and pathophysiological conditions related to neurodegeneration, biological nitration reactions, mainly deriving from the interaction between nitrogen monoxide (NO), its derivatives, and ROS, have been observed in vivo [10]. In particular, nitrated lipids, such as 5-nitro-γ-tocopherol, and nitrated proteins containing 3-nitrotyrosine have been found in AD brains [10,11]. Protein nitration represents an early event in neurodegenerative pathologies since it is biologically selective and site-specific: it is worth noting that nitration of a single tyrosine residue can cause heavy changes in the hydrophobicity and electrostatic properties of the molecules [10,12,13]. Furthermore, protein nitration modulates the activity of enzymes involved in neurodegeneration, and it could also affect the protein aggregation properties (for example, a tendency to form Tyr–Tyr-containing oligomers has been reported) [14,15,16].

Among the redox active species of physiological relevance, it is necessary to consider heme (or hemin, when the iron ion in it is reduced, Fe^II^, or in its oxidized, Fe^III^, form, respectively), whose level increases in pathological conditions or traumatic brain injuries. Heme is essential for organisms, constituting heme-proteins and carrying out signaling functions, but it is also characterized by high toxicity due to its potential oxidizing properties [17] (for example, it has been reported that 3–30 µM hemin is sufficient to kill 60–70% of cultured neurons and astrocytes within 4–14 h [18,19]). Therefore, multiple control mechanisms operate to keep the concentration of free heme low [20,21,22]. Interestingly, an abnormal heme *b* concentration has been observed in the temporal brain of AD patients with up to a 2.5-fold increase with respect to controls [23]. Moreover, a recent Raman study suggests the accumulation of heme in the senile plaques of human samples, although the type of the heme (i.e., free heme or from a hemoprotein) is still unclear [24]. The dysfunction of regulatory heme pathways, hemin complexation, by Aβ peptides and its subsequent pro-oxidase effect have been proposed to play a role in AD progression. Indeed, it has been established by different research groups that Aβ peptides, both those present in vivo in the human brain, Aβ_40_ and Aβ_42_, and fragments, such as Aβ_16_, or their mutants, can interact with hemin [23,25,26,27]. In particular, hemin *b* can coordinate up to two Aβ_16_ molecules, thus establishing an equilibrium between the low-spin hexa-coordinated complex [hemin(Aβ_16_)_2_] and the high-spin penta-coordinated complex [hemin(Aβ_16_)], which is strongly affected by peptide concentration and temperature [25]. Moreover, the coordination of peptides to hemin is reported in several studies to enhance its peroxidase activity [28,29,30], and this reactivity has also been reported in literature for the hemin–Aβ_16_ complex [1,27,31]. Although the link between nitration reactions and AD has been well established, the possibility that these biological nitrations are catalyzed by hemin–Aβ complexes has not yet been studied, and data on their pseudo-peroxidase activity in the presence of nitrite are still lacking. The interaction between these complexes and the nitrite anion has recently been studied taking into consideration the nitrite reductase activity only [32,33].

The aim of this study is, therefore, to deepen the investigation of peroxidase activity and the promotion of nitration reactions by the hemin–Aβ_16_ complex. The relevance of these reactions arises in the presence of heavy heme release caused by traumatic brain injuries or when slow but prolonged activity occurs since neurodegenerative diseases develop over many years [27,34]. We chose the Aβ_16_ fragment as the model of Aβ since it contains three histidines, which could provide axial coordination to iron ions, and has a lower tendency to aggregate than Aβ_40_ and Aβ_42_. The activation effect exerted by the coordination of the Aβ_16_ peptide on the hemin activity has been investigated considering the ability of the hemin–Aβ_16_ complex to catalyze oxidation and nitration reactions in the presence of hydrogen peroxide and nitrite (or in some cases of the peroxynitrite anion, ONOO^−^), exploiting both biologically relevant phenols and catechols and the Aβ_16_ peptide itself as target substrates.

## 2. Materials and Methods

### 2.1. Instruments

UV-Vis spectra were recorded on an Agilent (Santa Clara, CA, USA) 8453 diode array spectrophotometer equipped with a magnetically stirred quartz optical cell of 1 cm path length. Peptide purification was performed on a Shimadzu HPLC instrument equipped with two LC-20AD pumps and an SPDM20A diode array detector (working range: 190–800 nm) using a Phenomenex Jupiter 4U Proteo semipreparative column (4 μm, 250 × 10 mm). Mass spectrometry analysis was performed on an LCQ ADV MAX ion-trap mass spectrometer with an ESI ion source. The ESI conditions were as follows: capillary temperature 210 °C, tube lens voltage −25 V, and source voltage +4.9 kV. The system was run in automated LC-MS/MS mode, using a surveyor HPLC system (Thermo Finnigan, San Jose, CA, USA) equipped with a Phenomenex Jupiter 4U Proteo column (4 μm, 150 × 2.0 mm). For the analysis of peptide fragments, Bioworks 3.1 and Xcalibur 2.0.7 SP1 software were used (Thermo Finnigan, San Jose, CA, USA).

### 2.2. Materials

Protected amino acids, rink amide resin, and other reagents for peptide synthesis were purchased from Novabiochem. All other reagents were from Sigma-Aldrich or Merck.

### 2.3. Peptide Synthesis

Aβ_16_ (_1_DAEFRHDSGYEVHHQK_16_-NH_2_) was synthesized using traditional fluorenyl methoxy-carbonyl (Fmoc) solid-phase synthesis in DMF, as previously reported [35,36,37]. A rink-amide resin MBHA (substitution 0.52 mmol/g) was used as polymeric support in order to obtain amidation at the peptide C-terminus. At the end of the synthesis, the peptide was released from the resin and purified by HPLC, as previously reported [35].

### 2.4. Stock Solutions

Hemin stock solutions were prepared by dissolving 2–3 mg of hemin in 1 mL of NaOH 0.1 M, sonicating for 1 h, and centrifuging at 14,000 rpm for 5 min. The precipitate was eliminated, and sonication and centrifugation were repeated at least once until no precipitate was formed. The stock solution was stored at −18 °C for one week, and every day, an aliquot was diluted 1:10 in Milli-Q water and quantified spectrophotometrically using the molar extinction coefficient ε_390_ = 64,700 M^−1^ cm^−1^ (determined by the hemochromogen assay [38]), obtaining a 200–250 μM hemin concentration. Aβ_16_ solutions were prepared by dissolving 2–3 mg of lyophilized peptide in 1 mL of Milli-Q water. The solutions were quantified spectrophotometrically using the molar extinction coefficient ε_280_ = 1480 M^−1^ cm^−1^ [39] and stored at −18 °C for a couple of weeks. Phosphate buffer 100 mM at pH = 7.4 was prepared by dissolving the appropriate amount of NaH_2_PO_4_ and Na_2_HPO_4_ solid salts in Milli-Q water. The pH was adjusted by adding droplets of an aqueous concentrated NaOH solution. The substrate (3-(4-hydorxyphenil)propanoic acid (HPA, 25 mM), tyramine (Tym, 25 mM), L-tyrosine (L-Tyr, 1 mM for its reduced solubility in water), dopamine (DA, 25 mM), L-DOPA (25 mM), and NaNO_2_ (10 mM and 3 M) solutions were prepared by dissolving exact quantities of the solid compounds in phosphate buffer 100 mM at pH = 7.4. The pH of the solutions was controlled and, when required, adjusted by adding droplets of aqueous concentrated NaOH or H_3_PO_4_ solutions. The nitrite solutions were stored at room temperature to prevent precipitation; all the other solutions were stored at 4 °C. 

### 2.5. Kinetic Studies

The oxidation and nitration reactions were followed spectrophotometrically by observing the development of the absorption band of the reaction products, as previously reported [40]. All experiments were performed in phosphate buffer 100 mM at pH = 7.4.

The molar extinction coefficients of the reaction products, are the following: (1) oxidated products: for HPA (α-α dimer and Pummerer’s ketone) ε_300_ = 1950 M^−1^ cm^−1^ [41], for Tym (α-α dimer) ε_300_ = 1460 M^−1^ cm^−1^ [41], for L-Tyr (α-α dimer) ε_300_ = 1350 M^−1^ cm^−1^ [41], for DA (dopaminochrome) ε_476_ = 3300 M^−1^ cm^−1^ [41], and for L-DOPA (dopaquinone and dopachrome) ε_476_ = 3600 M^−1^ cm^−1^ [42]; (2) nitrated products: for HPA (3-nitroHPA) ε_450_ = 3350 M^−1^ cm^−1^ [40], for Tym (3-nitrotyramine) ε_450_ = 2300 M^−1^ cm^−1^ [40], for L-Tyr (3-nitrotyrosine) ε_450_ = 3100 M^−1^ cm^−1^ [40], for DA (6-nitrodopamine, 6NDA) ε_422_ = 2380 M^−1^ cm^−1^, and L-DOPA (nitroDOPA) was used the same ε_422_ as for DA. The ε_422_ of 6NDA was determined spectrophotometrically by recording the UV-Vis absorption spectrum of a solution containing an exact quantity of 6NDA in phosphate buffer at pH 7.4. 6NDA was synthetized by nitration of DA: a solution of 25 mg of DA, solubilized in 10 mL of Milli-Q water and added with 5 equivalents of NaNO_2_ and a drop of concentrated H_2_SO_4_, was left to react for 10 min in an ultrasound bath; 6NDA was then purified by HPLC (in isocratic conditions with Milli-Q water containing 0.1% of trifluoroacetic acid), lyophilized, resuspended with water and HCl, and lyophilized again; 6NDA was obtained and stored as hydrochloride salt.

For both oxidation and nitration reactions of phenolic substrates (here below generically referred to as PhOH), the concentrations of hemin (2 μM) and Aβ_16_ (10 μM) were kept constant. The concentrations of the other reactants were varied as follows: (1) oxidation reaction, dependence of the reaction rate on peroxide concentration: with HPA and Tym, [PhOH] = 3 mM and [H_2_O_2_] = 0.5–40 mM; with L-Tyr, [PhOH] = 0.7 mM and [H_2_O_2_] = 0.5–40 mM; dependence of the reaction rate on phenol concentration: with HPA, [PhOH] = 0.025–3 mM and [H_2_O_2_] = 20 mM; with Tym, [PhOH] = 0.1–4 mM and [H_2_O_2_] = 20 mM; with L-Tyr, [PhOH] = 0.01–0.9 mM and [H_2_O_2_] = 20 mM; (2) nitration reaction with H_2_O_2_/NO_2_^−^, dependence of the reaction rate on peroxide concentration: with HPA, [PhOH] = 3 mM, [NO_2_^−^] = 500 mM and [H_2_O_2_] = 2.5–100 mM; with Tym, [PhOH] = 3 mM, [NO_2_^−^] = 500 mM and [H_2_O_2_] = 1–100 mM; with L-Tyr, [PhOH] = 0.7 mM, [NO_2_^−^] = 200 mM and [H_2_O_2_] = 0.5–100 mM; dependence of the reaction rate on phenol concentration: with HPA, [PhOH] = 0.025–3 mM, [NO_2_^−^] = 500 mM and [H_2_O_2_] = 50 mM; with Tym, [PhOH] = 0.05–5 mM, [NO_2_^−^] = 500 mM and [H_2_O_2_] = 50 mM; with L-Tyr, [PhOH] = 0.01–0.9 mM, [NO_2_^−^] = 200 mM and [H_2_O_2_] = 30 mM; dependence of the reaction rate on nitrite concentration: with HPA, [PhOH] = 3 mM, [NO_2_^−^] = 40–700 mM and [H_2_O_2_] = 50 mM; with Tym, [PhOH] = 3 mM, [NO_2_^−^] = 5–500 mM and [H_2_O_2_] = 50 mM; with L-Tyr, [PhOH] = 0.7 mM, [NO_2_^−^] = 1–200 mM and [H_2_O_2_] = 30 mM; (3) nitration reaction with ONOO^−^, dependence of the reaction rate on phenol concentration: with HPA, [PhOH] = 0.01–6 mM and [ONOO^−^] = 0.6 mM; with Tym, [PhOH] = 0.01–1.5 mM and [ONOO^−^] = 0.6 mM; with L-Tyr, [PhOH] = 0.005–0.6 mM and [ONOO^−^] = 0.1 mM; dependence of the reaction rate on peroxinitrite concentration: with HPA, [PhOH] = 0.5 mM and [ONOO^−^] = 0.01–0.6 mM; with Tym, [PhOH] = 1 mM and [ONOO^−^] = 0.2–1.2 mM; with L-Tyr, [PhOH] = 0.15 mM and [ONOO^−^] = 0.03–0.2 mM.

For both oxidation and nitration reactions of catechol substrates (here below generically referred to as cat), the concentrations of hemin (0.2 μM) and Aβ_16_ (1 μM) were kept constant. The concentrations of the other reactants were varied as follows: (1) oxidation reaction, dependence of the reaction rate on peroxide concentration: with DA and L-DOPA, [cat] = 3 mM and [H_2_O_2_] = 10–700 mM; dependence of the reaction rate on catechol concentration: with DA, [cat] = 0.05–3 mM and [H_2_O_2_] = 500 mM; with L-DOPA [cat] = 0.05–3.5 mM and [H_2_O_2_] = 200 mM; (2) nitration reaction with H_2_O_2_/NO_2_^−^, dependence of the reaction rate on peroxide concentration: with DA, [cat] = 3 mM, [NO_2_^−^] = 500 mM and [H_2_O_2_] = 10–500 mM; with L-DOPA, [cat] = 3 mM, [NO_2_^−^] = 500 mM and [H_2_O_2_] = 10–700 mM; dependence of the reaction rate on catechol concentration: with DA, [cat] = 0.05–3 mM, [NO_2_^−^] = 500 mM and [H_2_O_2_] = 500 mM; with L-DOPA, [cat] = 0.05–3.8 mM, [NO_2_^−^] = 500 mM and [H_2_O_2_] = 400 mM; dependence of the reaction rate on nitrite concentration: with DA, [cat] = 3 mM, [NO_2_^−^] = 20–500 mM and [H_2_O_2_] = 500 mM; with L-DOPA, [cat] = 3 mM, [NO_2_^−^] = 1–500 mM and [H_2_O_2_] = 400 mM; (3) nitration reaction with ONOO^−^, dependence of the reaction rate on catechol concentration: with DA, [cat] = 0.1–5 mM and [ONOO^−^] = 1 mM; with L-DOPA, [cat] = 0.1–3 mM and [ONOO^−^] = 0.6 mM; dependence of the reaction rate on peroxinitrite concentration: with DA, [cat] = 3 mM and [ONOO^−^] = 0.05–1.8 mM; with L-DOPA, [cat] = 3 mM and [ONOO^−^] = 0.1–1.5 mM.

### 2.6. Mass Spectrometry Studies

The samples for the peptide modifications analysis were prepared by mixing hemin (2 μM), Aβ_16_ (10 μM), and, when needed, the substrate (3 or 0.3 mM) in phosphate buffer 100 mM at pH 7.4. For the analysis of hemin modifications, solutions of hemin (20 μM) and Aβ_16_ (100 μM) in phosphate buffer 100 mM at pH 7.4 were used. Hydrogen peroxide and nitrite were then added in 5 aliquots of 40 μM each (mild conditions) or 4 mM H_2_O_2_/40 mM NO_2_^−^ (harsh conditions) every 5 min. The samples were then incubated at 37 °C for 30 min before injection in the mass spectrometer. The elutions were carried out with Milli-Q water added with 0.1% of formic acid (solvent A) and acetonitrile added with 0.1% of formic acid (solvent B), with a flow rate of 0.2 mL/min. The solvent gradient started with 98% solvent A for 5 min, followed by a linear gradient from 98% to 55% solvent A in 65 min and 0% solvent A in 40 min for the analysis of peptide and hemin modifications, respectively.

## 3. Results and Discussion

The aim of this work was to investigate the reactivity of the hemin–Aβ_16_ complex, taking into consideration its pseudo-peroxidase activity in both oxidation and nitration reactions. Regarding the latter, previous studies show that heme-containing proteins, both peroxidase enzymes and proteins with other physiological functions, such as myoglobin, can catalyze tyrosine nitration in the presence of hydrogen peroxide and nitrite [40,43,44,45]. The proposed reaction mechanism involves several steps and can be described by the following reactions [40]:

PFe^III^ + H_2_O_2_ → ^+•^PFe^IV^ = O + H_2_O, (1)

^+•^PFe^IV^ = O + NO_2_^−^ → [^+•^PFe^IV^ = O/NO_2_^−^], (2)

[^+•^PFe^IV^ = O/NO_2_^−^] → PFe^IV^ = O + NO_2_^•^, (3)

^+•^PFe^IV^ = O + PhOH → [^+•^PFe^IV^ = O/PhOH], (4)

[^+•^PFe^IV^ = O/PhOH] → PFe^IV^ = O +PhO^•^ + H^+^, (5)

PFe^IV^ = O + NO_2_^−^ → [PFe^IV^ = O/NO_2_^−^], (6)

[PFe^IV^ = O/NO_2_^−^] + 2 H^+^ → PFe^III^ + NO_2_^•^ + H_2_O, (7)

PFe^IV^ = O + PhOH → [PFe^IV^ = O/PhOH], (8)

[PFe^IV^ = O/PhOH] + H^+^ → PFe^III^ + PhO^•^ + H_2_O, (9)

NO_2_^•^ + PhO^•^ → O_2_N-Ph-OH, (10)

NO_2_^•^ + PhOH → PhO^•^ + NO_2_^−^, (11)

2 PhO^•^ → dimers, (12)

2 NO_2_^•^ + H_2_O → NO_2_^−^ + NO_3_^−^ + 2 H^+^. (13)

In the first step, the coordination of hydrogen peroxide to the iron ion leads to the formation of the oxoferryl species ^+•^PFe^IV^ = O (where P indicates the porphyrin ring), containing a radical cation localized on the porphyrin or the polypeptidic chain and similar to peroxidases Compound I (Reaction 1), and the elimination of a water molecule. The reaction of the ^+•^PFe^IV^ = O species with either nitrite (Reactions 2 and 3) or a phenolic substrate, here again generically referred to as PhOH (Reactions 4 and 5), generates NO_2_^•^ or a phenoxy radical (PhO^•^) and the oxoferryl species PFe^IV^ = O, similar to peroxidases Compound II, by one-electron oxidation. This species is less reactive than the previous intermediate and can react either with nitrite (Reactions 6 and 7) or with the phenol (Reactions 8 and 9), forming the respective aforementioned radicals and acquiring two protons, restoring the initial PFe^III^ species with the elimination of a second water molecule. NO_2_^•^ and the phenoxy radicals can then couple, forming a nitro-phenol derivative, O_2_N-Ph-OH (Reaction 10). NO_2_^•^ can also react with a phenol, generating a phenoxy radical and restoring nitrite (Reaction 11) and can also undergo dismutation forming nitrite and nitrate (Reaction 13). The reaction between the phenoxy radicals in solutions generates phenolic dimers as byproducts (Reaction 12) [40].

Our experimental data (see below) suggest a similar catalytic cycle for the nitration reactions catalyzed by the hemin–Aβ_16_ complex.

In addition to the H_2_O_2_/NO_2_^−^ system, we also considered an alternative nitrating agent, ONOO^−^, which in vivo is formed by the reaction of NO^•^ with a superoxide anion (O_2_^•−^) (Reaction 14). Peroxynitrite has been reported to perform various biologically relevant oxidation reactions, but it can also nitrate aromatic compounds, both alone and in the presence of metal complexes [46].

NO^•^ + O_2_^•−^ → ONOO^−^. (14)

**Figure 1:**
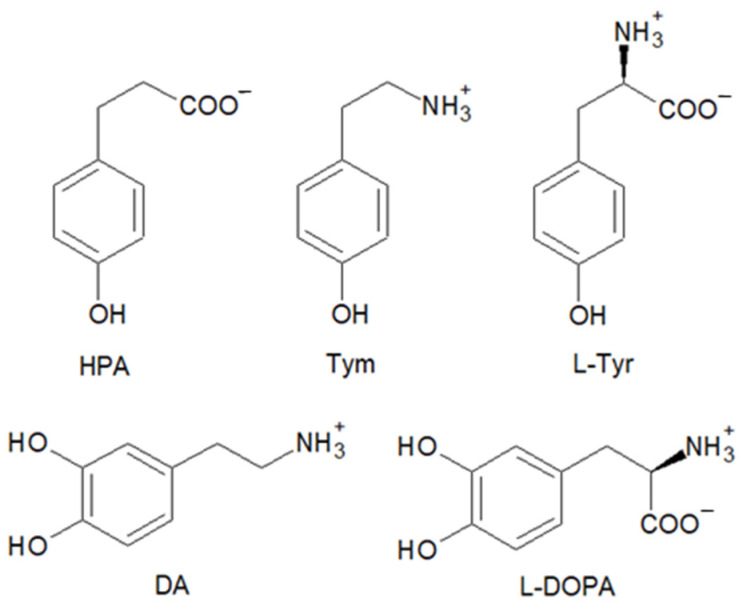
Structure of the substrates used in the kinetic and mass spectrometry studies: 3-(4-hydroxyphenyl)propanoic acid (HPA), tyramine (Tym), L-tyrosine (L-Tyr), dopamine (DA), and L-DOPA.

To study the ability of the hemin–Aβ_16_ complex to catalyze oxidation and nitration reactions, we exploited a selection of biologically relevant phenolic and catechol substrates: some of them are present in the brain, and others possess a similar structure but differ in their net charge or redox potential. In particular, we used HPA, Tym, and L-Tyr as phenols and DA and L-DOPA as catechols (Figure 1).

### 3.1. Kinetic Studies

#### 3.1.1. Hemin–Aβ_16_ Ratio

**Figure 2:**
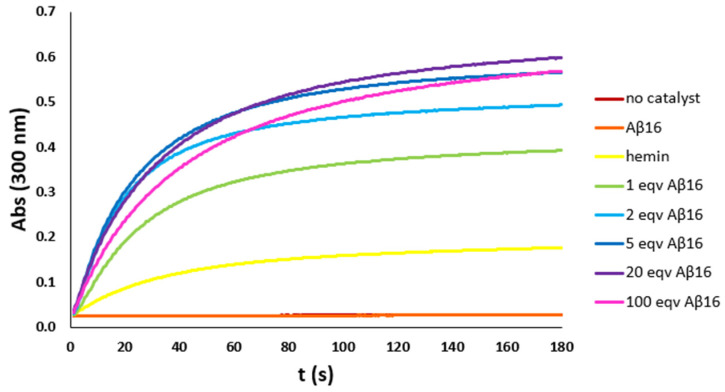
Kinetic trends of HPA (3 mM) oxidation with H_2_O_2_ (20 mM) in phosphate buffer (100 mM, pH 7.4) at 25 °C, with no catalyst (brown trace) and in the presence of hemin (2 μM) (yellow trace), Aβ_16_ (2 μM) (orange trace), or hemin–Aβ_16_ complex with increasing amounts of Aβ_16_ (1 eqv green trace, 2 eqv light blue trace, 5 eqv blue trace, 20 eqv purple trace, and 100 eqv pink trace). Both the trends with no catalyst and in the presence of only the peptide are flat, indicating no activity.

As previously reported, Aβ_16_ coordinates hemin with a stoichiometry that is temperature and peptide concentration dependent. So, to optimize the hemin:Aβ_16_ ratio, a set of kinetic studies on the pseudo-peroxidase activity of the hemin–Aβ_16_ complex with increasing amounts of the peptide was performed, with the phenol HPA (Figure 2) and the catechol DA (Figure S1) as models for the other phenols and catechols used in further kinetic studies. The kinetic studies were performed with different hemin concentrations depending on the substrate employed, i.e., 2 μM with phenols and 0.2 μM with catechols, respectively, due to the much higher catalytic activity of hemin towards the latter substrates.

The kinetic trends show that peroxidase activity is absent without hemin and that the moderate catalytic activity of hemin increases by adding the peptide up to five equivalents. With higher amounts of peptide, the catalytic activity persists, but the initial reaction rate slightly decreases: above six equivalents of peptide, the less active hexa-coordinated hemin accumulates in solution. So, the hemin:Aβ_16_ ratio of 1:5 was chosen for further analysis.

#### 3.1.2. Oxidation Reaction

The kinetic constants (Table 1) were determined as follows. Since the reaction rate depends on both the hydrogen peroxide and substrate concentrations, at first, the hydrogen peroxide concentration was varied in a low concentration range, fixing that of the substrate at a saturation value so that Reaction (1) becomes the rate-determining step of the catalytic cycle (in the absence of nitrite only reactions 1, 4, 5, 8, 9, and 12 occur). k_1_, the second order kinetic constant related to Reaction (1), was estimated by fitting with a linear equation the reaction rate vs. [H_2_O_2_] trend at low [H_2_O_2_] values. Then, the substrate concentration was varied, fixing that of hydrogen peroxide at a saturating value, so Reactions (8) and (9) become the rate-determining steps of the cycle. A hyperbolic Michaelis–Menten trend was obtained, and k_cat_, the kinetic constants related to Reaction (9) and K_M_, the Michaelis–Menten constants related to the dissociation equilibrium between catalyst and substrate, were obtained (Figures S2–S6).

**Figure 3:**
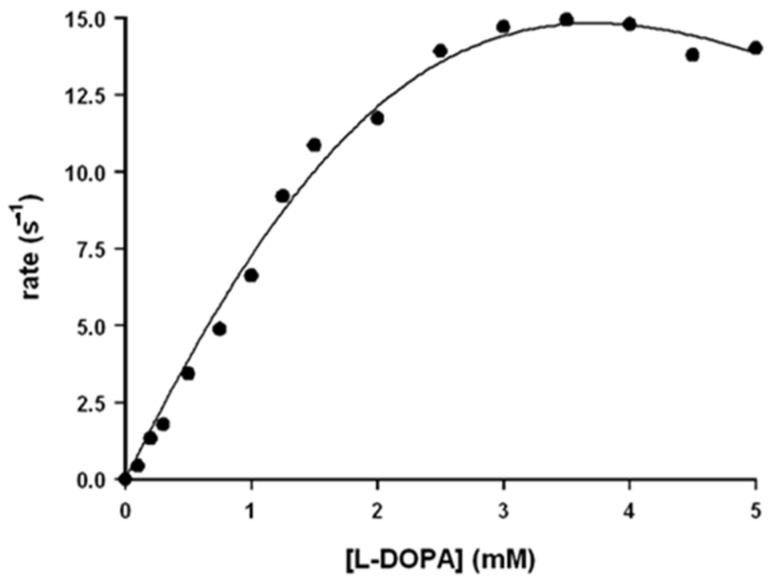
Rate dependence vs. [L-DOPA] for the oxidation reaction of L-DOPA in the presence of Aβ_16_ 1 μM and hemin 0.2 μM, in phosphate buffer 100 mM, pH 7.4 at 25 °C.

In the case of the rate dependence on L-DOPA concentration, the observed trend cannot be fitted with the Michaelis–Menten equation: the reaction rate increases upon increasing [L-DOPA] for low concentration and slightly decreases for high ones (Figure 3). This trend is probably due to the binding of two L-DOPA molecules to the catalyst, one near the hemin group and the other in another site, probably far from the hemin group. The experimental rate profile was fitted with Equation (15), in which the reaction rate depends on two different k_cat_ values, here indicated as k_c1_ and k_c2_ (the first one related to the electron transfer when one substrate molecule is bound and the second one for the electron transfer when two substrate molecules are bound), and also on two different K_M_ values, here indicated as K_B1_ and K_B2_ (related to the stepwise binding equilibria of the two L-DOPA molecules and that could be approximated as K_B1_ = 1/K_M1_ and K_B2_ = 1/K_M2_).

(15) $$\mathrm{rate}=\frac{k_{c1}\times K_{B1}\times \left[S\right]+k_{c2}\times K_{B1}\times K_{B2}\times {\left[S\right]}^{2}}{1+K_{B1}\times \left[S\right]+K_{B1}\times K_{B2}\times {\left[S\right]}^{2}}.$$

The use of the multiparameters Equation (15) leads to kinetic constants with high standard deviations so that for L-DOPA, only k_c1_, K_M1_, and k_c1_/K_M1_ could be obtained with good precision (Table 1).

#### 3.1.3. Nitration Reaction with the H_2_O_2_/NO_2_^−^ System

The nitration reaction was investigated in the presence of the H_2_O_2_/NO_2_^−^ system as a nitrating agent, and, after preliminary tests performed with increasing amounts of peptide (similar to those above mentioned for the oxidation reaction) (Figures S7 and S8), the kinetic constants were determined (Table 2). Since in the nitration reaction, there are two substrates which can interact with the catalyst (the phenol/catechol and the nitrite anion), two different K_M_ and k_cat_ constants can be determined, $K_{M}^{S}\;\mathrm{and}\;k_{\mathrm{cat}}^{S}$, related to the phenolic/catechol substrate, and $K_{M}^{\mathrm{nitrite}}\;\mathrm{and}\;k_{\mathrm{cat}}^{\mathrm{nitrite}}$, related to NO_2_^−^, respectively [40]. In detail, we proceeded as previously described: at first, the hydrogen peroxide concentration was varied, fixing those of the substrate and nitrite at a saturating value so that Reaction (1) becomes the rate-determining step of the catalytic cycle, and k_1_ was estimated from the rate dependence vs. [H_2_O_2_]. Then, the aromatic substrate concentration was varied, fixing those of hydrogen peroxide and nitrite at a saturating value so that Reaction (9) becomes the rate-determining step of the cycle. Michaelis–Menten trends were obtained, and the kinetic constants $K_{M}^{S}\;{\mathrm{and}\;k}_{\mathrm{cat}}^{S}$ were estimated. Finally, the nitrite concentration was varied, fixing those of hydrogen peroxide and of the substrate at a saturating value so that Reaction (7) becomes the rate-determining step of the cycle. In most cases, Michaelis–Menten trends were obtained, and the kinetic constants $K_{M}^{\mathrm{nitrite}}\;\mathrm{and}\;k_{\mathrm{cat}}^{\mathrm{nitrite}}$ were estimated (Figures S9–S13).

**Figure 4:**
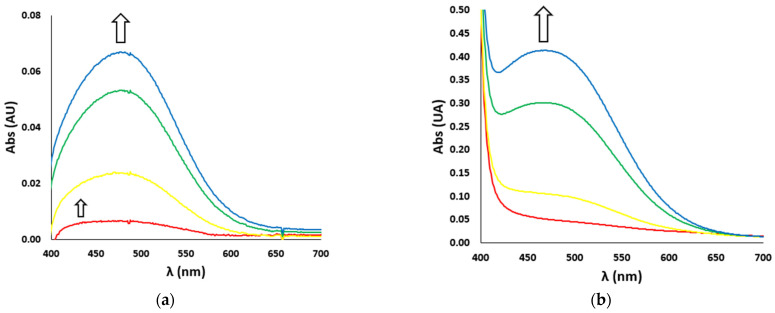
UV-Vis spectra vs. time (red line t = 0 s, yellow line t = 10 s, green line t = 60 s, blue line t = 120 s) of (**a**) nitration reaction with the H_2_O_2_/NO_2_^−^ system (H_2_O_2_ 500 mM and NO_2_^−^ 500 mM) and (**b**) oxidation reaction (with H_2_O_2_ 100 mM) of DA (3 mM), in the presence of Aβ_16_ 1 μM and hemin 0.2 μM, in phosphate buffer 100 mM, pH 7.4 at 25 °C. The smaller (**a**) and bigger (**a**,**b**) arrows indicate the development over time of the absorption bands of the nitrated (422 nm) and oxidated (476 nm) products, respectively.

In the case of catechol nitration, the UV-Vis spectra show two different bands (Figure 4): during the very first seconds of reaction, an absorption band at 422 nm, typical of the nitrated product, develops (Figure 4a, smaller arrow), while after a few seconds a much more intense band at 476 nm, typical of the oxidated product (i.e., dopaminochrome, Figure 4b), raises and covers the first one (Figure 4a, bigger arrow). In the kinetic studies, we considered the first instants of the reaction and the related band at 422 nm.

The majority of the kinetic traces exhibit Michaelis–Menten trends, as usually reported for heme-proteins as catalysts [40]. However, the rate dependence of HPA nitration on nitrite concentration shows a sigmoidal trend (Figure S9): this probably indicates that at low nitrite concentration, HPA dimerization (Reaction 12) competes with its nitration (Reaction 10) due to an accumulation of phenoxy radicals in solution. In the rate dependence of L-DOPA nitration on nitrite concentration, another trend consisting of an increasing rate at low and a decreasing rate at high nitrite concentration, respectively, is observed (Figure S13). This trend could be explained assuming that different nitration mechanisms coexist as occurs with heme enzymes and proteins [40,44,45].

Both for oxidation and nitration reactions, k_1_ should be independent of the substrate since it is related to Reaction (1) and involves the activation of hydrogen peroxide by hemin. However, the different k_1_ values obtained and reported in Table 1 and Table 2 indicate that phenolic and catechol substrates may play a role in activating hydrogen peroxide through acid/base catalysis. It has been observed that the presence of acidic or basic residues in the active site pocket of peroxidases (for example, a His and an Arg in HRP) assists the formation of the intermediates of the catalytic cycle [47].

Regarding the kinetic constants obtained for the nitration reaction, the k_1_ and k_cat_ values are lower than the ones obtained for the oxidation reaction. This is because NO_2_^•^ produced by Reaction (3) is also involved in the unproductive dismutation to NO_2_^−^ and NO_3_^−^ (Reaction 13).

Analysis of the kinetic constants reported in Table 1 and Table 2 (for oxidation and nitration reactions, respectively) also highlights that for each substrate, the K_M_ value obtained for its oxidation is in the same order of magnitude of the $K_{M}^{S}$ value obtained for its nitration, and that $K_{M}^{S}$ is much higher than $K_{M}^{\mathrm{nitrite}}$. This indicates that the PFe^IV^ = O species reacts preferentially with phenols or catechols instead of nitrite, probably through a π–π electron transfer between the aromatic systems. The slight difference between K_M_ and $K_{M}^{S}$ for each substrate is due to the nitrite saturating concentration in the latter case, with the nitrite anion probably also bound to the Aβ peptide.

Finally, it can be noted that, for both oxidation and nitration reactions, the K_M_ values increase from HPA to Tyr and from Tyr to Tym: this indicates that the catalytic system binds preferentially negatively charged substrates than positively charged ones (or containing a positive charge).

#### 3.1.4. Nitration Reaction with ONOO^−^

Nitration in the presence of ONOO^−^ as a nitrating agent was also investigated, determining the kinetic constants reported in Table 3. After preliminary tests conducted with increasing amounts of peptide (Figures S7 and S8), we proceeded as follows: at first, the substrate concentration was varied, fixing [ONOO^−^] at a saturating value, and the $K_{M}^{S}\;\mathrm{and}\;k_{\mathrm{cat}}^{S}$ constants were estimated when a Michaelis–Menten trend was obtained (i.e., for all phenolic/catechol substrates except for HPA). Then, the peroxynitrite concentration was varied, fixing [substrate] at a saturating value: in this case, the $K_{M}^{\mathrm{peroxynitrite}}\;\mathrm{and}\;k_{\mathrm{cat}}^{\mathrm{peroxynitrite}}$ constants were estimated only for Tym, which exhibits a Michaelis–Menten trend (Figures S14–S18).

The UV-Vis spectra recorded during catechols nitration with ONOO^−^ show the absorption band at 422 nm, indicating that the corresponding nitrocatechols were the only products and that neither dopaminochrome nor dopaquinone was significantly formed.

The rate dependencies of DA and L-DOPA nitration on peroxynitrite concentration show sigmoidal behavior. Additionally, HPA and L-Tyr nitrations show a trend which cannot be fitted with the Michaelis–Menten equation: the reaction rate increases for low ONOO^−^ concentrations and decreases for high ones. This could be due to the non-negligible amount of nitrite (deriving from the peroxynitrite synthesis process); it firstly promotes the oxidation of the substrate to its radical form, which can subsequently evolve to the nitro-compound. With high concentrations of peroxynitrite, the high amount of the substrate radical makes competitive oxidation the predominant reaction.

The analysis of the nitration reaction promoted by peroxynitrite will require further studies, as the reaction mechanism is currently not known in detail.

### 3.2. Mass Spectrometry Studies

Mass spectrometry studies were performed to detect and quantify the modifications undergone by the peptide residues in the presence of the reactive species generated by the hemin/H_2_O_2_/NO_2_^−^ system. In particular, it was reported that Aβ_16_ can undergo three types of modifications in the presence of ROS/RNS: oxidation due to the binding of an oxygen atom to a His residue, nitration due to the substitution of a hydrogen atom with a nitro group on a Tyr residue, and dimerization due to the formation of a radical on a Tyr residue, followed by a coupling step [48,49,50]. Moreover, in the presence of catechols such as DA or 4-methylcatechol, the corresponding quinones can react with Cys, His, or Lys residues, forming adducts with the peptide [42,51].

**Figure 5:**
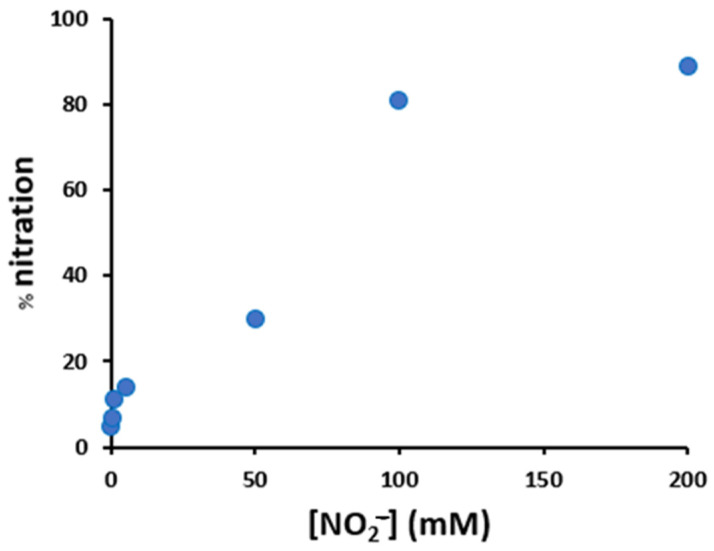
Variation of the nitration percentage of the peptide (10 μM), in the presence of hemin (2 μM), as a function of the concentration of nitrite and hydrogen peroxide (both varying from 0.2 to 200 mM), in phosphate buffer 100 mM, pH 7.4 after 30 min incubation at 37 °C.

The analysis of peptide modification was first performed with increasing amounts of hydrogen peroxide and nitrite (Figure 5 and Figure S19), and two conditions were then chosen for further analysis, 200 μM H_2_O_2_/200 μM NO_2_^−^ (mild conditions) and 20 mM H_2_O_2_/200 mM NO_2_^−^ (harsh conditions, analogous to those employed in the kinetic studies).

To evaluate which types of modification occurred and whether the presence of hemin and the concentration of the H_2_O_2_/NO_2_^−^ system influenced the peptide modification yield, eight samples were prepared and analyzed in HPLC-MS/MS, obtaining the results reported in Table 4.

**Figure 6:**
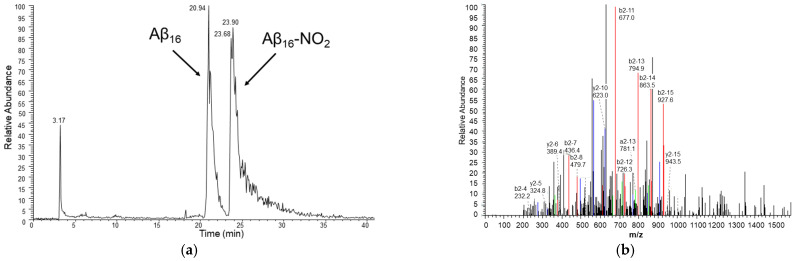
(**a**) HPLC chromatogram of a sample containing hemin 2 μM, Aβ_16_ 10 μM, hydrogen peroxide 20 mM and nitrite 200 mM, in phosphate buffer 100 mM, pH 7.4 at 25 °C and (**b**) MS/MS spectrum of the peak assigned to the Aβ_16_ peptide containing the nitrated Tyr_10_ residue (peptide mass of 1999 amu, corresponding to a mass increase of 45 amu with respect to the unmodified peptide). The assignment of the y (blue) and b (red) ion series (in double-charged states) is shown.

Considering the amino acid sequence of the Aβ_16_ peptide, i.e., DAEFRHDSGYEVHHQK, the expected sites of modifications are the tyrosine residue, Tyr_10_, that can undergo nitration, and the histidine residues, His_6_, His_13_, and His_14_, that can undergo oxidation. Figure 6 shows that nitration on Tyr_10_ is the principal modification; therefore, it was chosen as a marker of Aβ_16_ modification for further analysis. Oxidation of a histidine residue (without the possibility to distinguish among His_6_, His_13_, or His_14_) was also observed, but with very small yields. Since previous studies on the hemin–Aβ_16_ complex in oxidizing systems revealed the formation of Tyr–Tyr peptide dimers [1,10], this potential product (also in its oxidized or nitrated forms) was also taken into consideration, but under our conditions, it was never observed.

**Figure 7:**
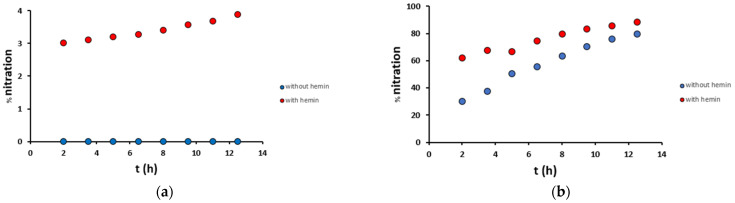
Percentage nitration of the Aβ_16_ peptide (10 μM) along time in (**a**) mild (200 μM H_2_O_2_/200 μM NO_2_^−^) and (**b**) harsh (20 mM H_2_O_2_/200 mM NO_2_^−^) conditions, in the presence (2 μM, red) and absence (blue) of hemin, in phosphate buffer 100 mM, pH 7.4 at 25 °C.

The time-dependent analysis of Aβ_16_ nitration, both in mild and harsh conditions and in the presence or absence of hemin, was performed at 90 min intervals (Figure 7). In mild conditions, in the absence of hemin, no modifications were observed, while in the presence of hemin, nitration on Tyr_10_ occurred with an increased yield from 3.0% after 2 h to 3.9% after 12.5 h. In harsh conditions, the yields were much higher: in the absence of hemin, nitration on Tyr_10_ occurred with an increasing yield from 30% after 2 h to 79% after 12.5 h, while in the presence of hemin from 62% after 2 h to 88% after 12.5 h. These data confirm that the presence of hemin enhances the reaction rates, doubling the nitration yield obtained after 2 h with respect to the not catalyzed reaction.

Further studies were performed adding a phenolic/catechol substrate to the reaction mixture to investigate its ability to protect the peptide from ROS/RNS damage. In particular, HPA, Tym, DA, or L-DOPA were employed (L-Tyr was not taken into consideration due to its low solubility) at two different concentrations and with the H_2_O_2_/NO_2_^−^ harsh conditions above reported (Table 5). It turned out that all the substrates exhibited a protecting effect: with L-DOPA, nitration at Tyr_10_ did not occur at all; with DA, it occurred with a very small yield, while with HPA and Tym, the nitration yields were higher (but still much lower than the 65.8% obtained without external substrates, Table 4). The observed protecting effect is related to the one-electron redox potentials of the substrates: E^0^ (DOPA) = 0.745 V [52] < E^0^ (DA) = 0.752 V [53] < E^0^ (HPA) = 1.007 V [54] < E^0^ (Tym) = 1.027 V [54]. The redox potential of Tyr residue inside a peptide is expected to be slightly lower than that of the free amino acid (E^0^ (Tyr) = 1.097 V [54]).

In the case of the reaction with catechols, the possible formation of adducts between catechols/quinones and His residues has also been taken into consideration, but these adducts were not observed in the HPLC-MS chromatograms.

Finally, the possible nitration reaction occurring on hemin was explored, considering that horse heart myoglobin treated with H_2_O_2_/NO_2_^−^ was reported to undergo hemin nitration by substitution of a proton from the porphyrin vinyl group with a NO_2_ group [40,48,55].

Our HPLC-MS analysis did not show the presence of nitrated hemin. Conversely, the chromatograms showed a decrease in the intensity of the hemin peaks, indicating a degradation process. Probably, the hemin coordination to a small peptide-like Aβ_16_ is not sufficient to protect the porphyrin from degradation, differently from what is observed with proteins such as myoglobin [48] or neuroglobin [43], where the protein backbone hinders access to the porphyrin ring.

**Figure 8:**
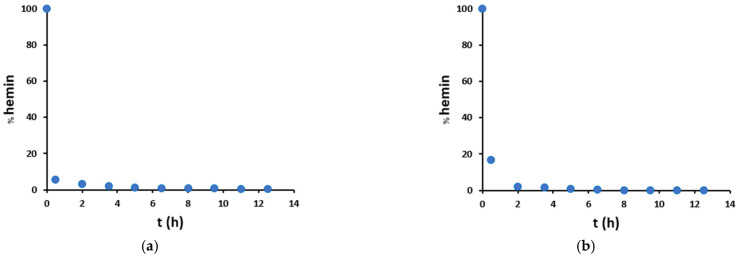
Percentage amount of unmodified hemin as compared to the blank sample without hydrogen peroxide, in the presence of peptide (100 μM) and hemin (20 μM), in (**a**) mild (200 μM H_2_O_2_/200 μM NO_2_^−^) and (**b**) harsh (20 mM H_2_O_2_/200 mM NO_2_^−^) conditions.

Therefore, hemin degradation promoted by H_2_O_2_/NO_2_^−^ was studied in more detail (Figure 8). Hemin signal decreases with time, starting from 100% in a blank sample (without hydrogen peroxide) and leading after 30 min to almost complete hemin degradation, both in mild and harsh conditions. Therefore, we can deduce that the catalyzed nitration of tyrosine residue runs out very quickly, and this is also in agreement with the trends observed in Figure 7: in the presence of hemin (red dots), there is a big increase in nitration yield from 0 to 2 h, and after that, there is just a small variation, due to both the not-catalyzed reaction and the catalysis given by free iron and degraded hemin. Instead, in the absence of hemin (blue dots), an almost linear increase trend is observed.

Furthermore, Figure 8 shows that in mild conditions, hemin degradation exhibits a higher initial rate than in harsh conditions. This is probably because nitrite partly protects hemin from degradation. Finally, in mild conditions, hemin degradation shows a hyperbolic trend over time (Figure 8a and Figure S20), while in harsh conditions, the hemin signal decreases much faster so that the catalyst is no more detectable after 8 h (Figure 8b and Figure S20).

## 4. Conclusions

The aim of this work was to investigate the ability of the hemin–Aβ_16_ complex to catalyze both oxidation and nitration reactions through a peroxidase-like mechanism since it has been established that the nitration reaction is related to AD onset, but to date, in literature, only the oxidative reactivity of the complex has been taken into consideration.

Our results indicate that hemin exhibits rich reactivity, which consists of not only oxidative but also nitrative catalytic activities. Moreover, it can modify both external aromatic substrates and bound peptides (such as Aβ). Our kinetic studies show that hemin’s reactivity is enhanced by Aβ_16_ coordination and let us estimate for the first time the kinetic parameters for both oxidation and nitration reactions catalyzed by the hemin–Aβ_16_ complex for a wide set of biologically relevant substrates. The estimated kinetic constants depend on the chemical structure and the substrate charge and let us gain information on the reactivity of the catalyst. Moreover, the mass spectrometry studies allowed us to deepen the investigation of the modifications undergone by the peptide itself because of the hemin–Aβ_16_ reactivity. In particular, we detected the targets (mainly Tyr_10_) and the entities of the modifications and highlighted that external substrates and the Aβ peptide only partly protect hemin from degradation so that the entity of the modifications depends on the environment: intact hemin possesses a great reactivity while the reactivity of degraded hemin is lower, but not completely quenched. These results clearly show that hemin contribution to redox reactivity in neurons strongly depends on the environment.

The kinetic constants obtained here can be compared to the data previously reported for analogous catalytic systems, and it can be noticed that the hemin–Aβ_16_ complex is, as expected, by far less active than natural peroxidases such as lactoperoxidase ($k_{\mathrm{cat}}^{\mathrm{PhOH}}$ = 380 ± 10 s^−1^ for the HPA nitration reaction in presence of H_2_O_2_ and NO_2_^−^) [48]. This result was easily predictable considering that our system contains a much smaller peptide component missing the primary contribution due to the presence of acid/base amino acid residues in the enzyme active site. The constants ($k_{\mathrm{cat}}^{\mathrm{PhOH}}$ = 0.308 ± 0.009 s^−1^ for the HPA nitration reaction in the presence of H_2_O_2_ and NO_2_^−^) are also lower than the ones exhibited by human myoglobin ($k_{\mathrm{cat}}^{\mathrm{PhOH}}$ = 2.1 ± 0.1 s^−1^ for the HPA nitration reaction in the presence of H_2_O_2_ and NO_2_^−^), which possesses a non-negligible catalytic contribution from the protein component [48], despite its natural function not being the catalytic one.

However, the reactivity of the hemin–Aβ_16_ complex must be taken into consideration in pathological conditions where an accumulation of free hemin occurs, for example, due to traumatic brain injuries: in this situation, the moderate activity of this system is enhanced due to an increase in the hemin concentration in the brain [56]. Moreover, in the presence of high amounts of the Aβ_16_ peptide, as occurs in the pathological conditions of AD, the cellular damages caused by ROS and RNS accumulations are enhanced. This small but significant effect of the hemin–peptide complex activity on the redox balance of neurons could become relevant considering the long-time development of AD.

## Figures and Tables

**Table 1:** Kinetic constants for the oxidation of phenolic/catechol substrates catalyzed by the hemin–Aβ_16_ complex in the presence of H_2_O_2_, in 100 mM phosphate buffer, pH 7.4 at 25 °C. (* Michaelis–Menten constant for the dissociation equilibrium of the first L-DOPA molecule, K_M1_; ** rate constant for the electron transfer when one L-DOPA molecule is bound, k_c1_; *** the value represents k_c1_/K_M1_).

Substrate	k_1_ (M^−1^ s^−1^)	K_M_ (M)	k_cat_ (s^−1^)	k_cat_/K_M_ (M^−1^ s^−1^)
HPA	116 ± 8	(2.1 ± 0.2) × 10^−4^	3.39 ± 0.08	(1.58 ± 0.12) × 10^4^
Tym	176 ± 3	(1.8 ± 0.4) × 10^−3^	4.9 ± 0.5	(2.7 ± 0.3) × 10^3^
L-Tyr	267 ± 15	(9.3 ± 1.9) × 10^−4^	4.0 ± 0.5	(4.4 ± 0.4) × 10^3^
DA	26.1 ± 1.3	(1.02 ± 0.11) × 10^−3^	14.1 ± 0.6	(1.39 ± 0.10) × 10^4^
L-DOPA	126 ± 16	(5.8 ± 1.7) × 10^−3^ *	47 ± 11 **	(8.2 ± 0.6) × 10^3^ ***

**Table 2:** Kinetic constants for the nitration of phenolic/catechol substrates catalyzed by the hemin–Aβ_16_ complex in the presence of H_2_O_2_ and NO_2_^−^, in 100 mM phosphate buffer, pH 7.4 at 25 °C (n.d. not determined).

Substrate	k_1_ (M^−1^ s^−1^)	$K_{M}^{S}$ (M)	$k_{cat}^{S}$ (s^−1^)	$K_{M}^{nitrite}$ (M)	$k_{cat}^{nitrite}$ (s^−1^)
HPA	10 ± 0.6	(1.8 ± 0.2) × 10^−4^	0.308 ± 0.009	0.26 ± 0.05	0.27 ± 0.02
Tym	28 ± 2	(1.13 ± 0.04) × 10^−3^	0.569 ± 0.007	0.053 ± 0.005	0.408 ± 0.011
L-Tyr	64 ± 2	(4.7 ± 0.5) × 10^−4^	1.91 ± 0.09	0.006 ± 0.001	1.03 ± 0.03
DA	21 ± 2	(3.2 ± 0.4) × 10^−4^	5.07 ± 0.17	0.033 ± 0.005	9.4 ± 0.3
L-DOPA	36 ± 6	(5.6 ± 0.8) × 10^−4^	11.2 ± 0.5	n.d.	n.d.

**Table 3:** Kinetic constants for the nitration of phenolic/catechol substrates catalyzed by the hemin–Aβ_16_ complex in the presence of ONOO^−^, in 100 mM phosphate buffer, pH 7.4 at 25 °C (n. d. not determined).

Substrate	$K_{M}^{S}$ (M)	$k_{cat}^{S}$ (s^−1^)	$K_{M}^{\mathrm{peroxynitrite}}$ (M)	$k_{cat}^{peroxynitrite}$ (s^−1^)
HPA	n. d.	n. d.	n. d.	n. d.
Tym	(2.8 ± 0.8) × 10^−4^	0.36 ± 0.03	(2.2 ± 0.4) × 10^−4^	0.69 ± 0.04
L-Tyr	(2.4 ± 0.5) × 10^−5^	2.02 ± 0.08	n. d.	n. d.
DA	(8.6 ± 0.5) × 10^−4^	356 ± 7	n. d.	n. d.
L-DOPA	(8.0 ± 0.6) × 10^−4^	323 ± 9	n. d.	n. d.

**Table 4:** Percentage oxidation (Aβ_16_-O) and nitration (Aβ_16_-NO_2_) of the peptide with or without hemin with hydrogen peroxide and nitrite at different concentrations.

[Aβ_16_]	[hemin]	[H_2_O_2_]	[NO_2_^−^]	Aβ_16_	Aβ_16_-O	Aβ_16_-NO_2_
10 μM	0 μM	200 μM	0 μM	99.7%	0.3%	-
10 μM	0 μM	200 μM	200 μM	97.7%	0.1%	2.2%
10 μM	0 μM	20 mM	0 mM	98.5%	1.5%	-
10 μM	0 μM	20 mM	200 mM	40.8%	0.7%	58.5%
10 μM	2 μM	200 μM	0 μM	99.7%	0.3%	-
10 μM	2 μM	200 μM	200 μM	91.2%	0.1%	8.7%
10 μM	2 μM	20 mM	0 mM	96.6%	3.4%	-
10 μM	2 μM	20 mM	200 mM	34.2%	0%	65.8%

**Table 5:** Percentage oxidation (Aβ_16_-O) and nitration (Aβ_16_-NO_2_) of the peptide in the presence of external substrates and Aβ_16_ 10 μM, hemin 2 μM, H_2_O_2_ 20 mM, and NO_2_^−^ 200 mM.

Exogenous Substrates		Aβ_16_	Aβ_16_-O	Aβ_16_-NO_2_
HPA	3 mM	98.5%	0%	1.5%
	0.3 mM	89.5%	0%	10.5%
Tym	3 mM	94.0%	0%	6.0%
	0.3 mM	67.3%	0%	32.7%
DA	3 mM	96.9%	2.3%	0.8%
	0.3 mM	95.8%	0.4%	3.8%
L-DOPA	3 mM	98.7%	1.3%	0%
	0.3 mM	99.2%	0.8%	0%

## Data Availability

Data supporting the reported results can be obtained from the corresponding author.

## Supplementary Materials

The following supporting information can be downloaded at: https://www.mdpi.com/article/10.3390/antiox12071319/s1, Additional kinetic traces (Figures S1, S7 and S8), trends of the initial rate of the oxidation (Figures S2–S6) and nitration (Figures S9–S18) reactions of the substrates, and percentage amount of nitrated peptide and unmodified hemin (Figures S19 and S20) are available online.
