# Supplementary material for: Biological Oxidations and Nitrations Promoted by the Hemin–Aβ16 Complex
Source: Antioxidants (Basel). 2023 Jun 21;12(7):1319. doi: 10.3390/antiox12071319 (PMC10376006; doi:10.3390/antiox12071319)
Supplement: Supplementary file 1 [file antioxidants-12-01319-s001.zip › antioxidants-2433060-supplementary.pdf]

Biological oxidations and nitrations promoted by hemin-Aβ_{16} complex

Silvia De Caro[1,2], Giulia De Soricellis[1], Simone Dell'Acqua[1], Enrico Monzani[1,*] and Stefania Nicolis[1,*]

[1] Dipartimento di Chimica, Università di Pavia, via Taramelli 12, 27100 Pavia, Italy; silvia.decaro01@universitadipavia.it (S.D.C.); giulia.desoricellis@unipv.it (G.D.S.); simone.dellacqua@unipv.it (S.D.); enrico.monzani@unipv.it (E.M.); stefania.nicolis@unipv.it (S.N.)

[2] Scuola Universitaria Superiore IUSS, piazza della Vittoria 15, 27100 Pavia, Italy

[*] Correspondence: enrico.monzani@unipv.it (E.M.); stefania.nicolis@unipv.it (S.N.)

Supporting information

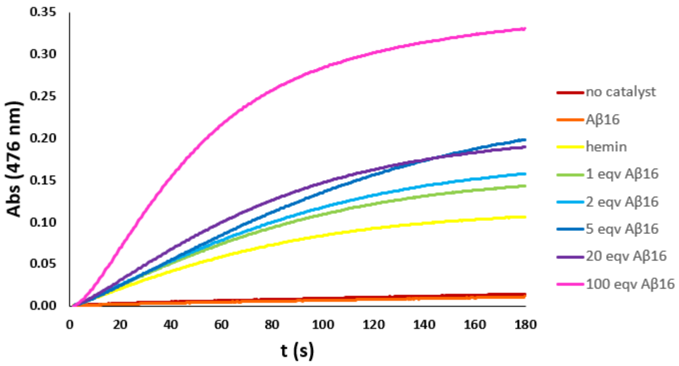

Figure S1: *kinetic trends of DA (3 mM) oxidation with H_2O_2 (50 mM) in phosphate buffer (100 mM, pH 7.4) at* 25 °C*, with no catalyst (brown trace) and in the presence of hemin (0.2 µM) (yellow trace), Aβ_{16} (0.2 µM) (orange trace) or hemin-Aβ_{16} complex with increasing amounts of Aβ_{16} (1 eqv green trace, 2 eqv light blue trace, 5 eqv blue trace, 20 eqv purple trace, 100 eqv pink trace). Both the trends with no catalyst and in presence of the peptide only are almost flat, indicating no activity.*

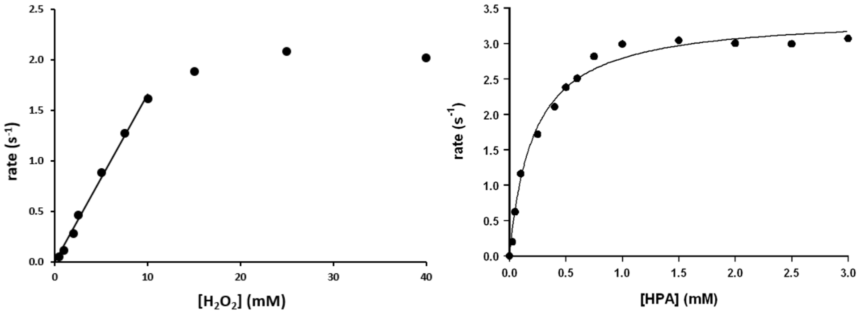

Figure S2: *trend of the initial rate of the oxidation reaction of the substrate HPA, in presence of Aβ_{16} 10 µM and hemin 2 µM, vs [H_2O_2] (left) and vs [HPA] (right), in phosphate buffer 100 mM, pH 7.4 at* 25 °C.

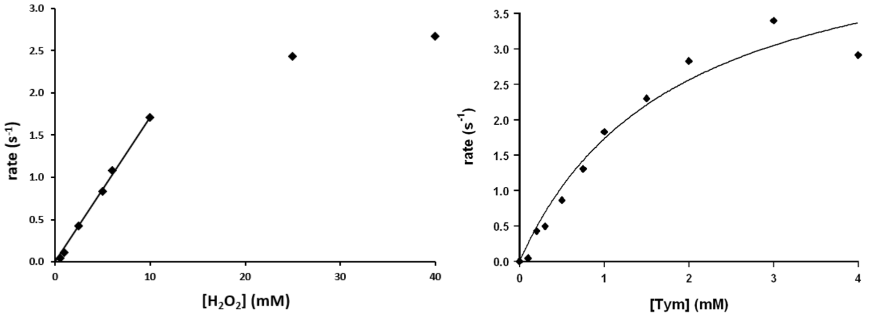

Figure S3: *trend of the initial rate of the oxidation reaction of the substrate Tym, in presence of* $A\beta_{16}$ *10* μM *and hemin 2* μM*, vs* $[H_2O_2]$ *(left) and vs [Tym] (right), in phosphate buffer 100 mM, pH 7.4 at* 25 °C.

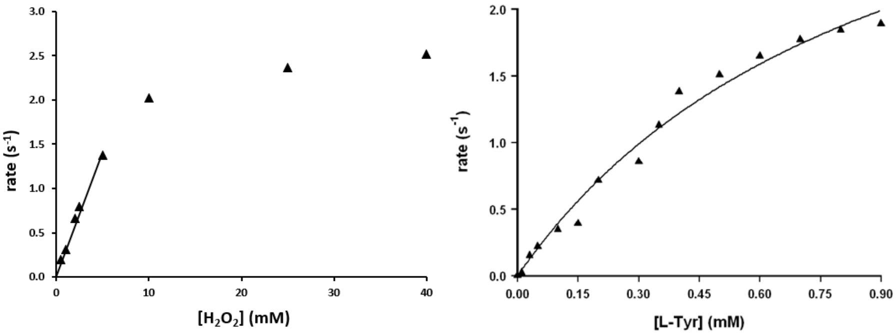

Figure S4: *trend of the initial rate of the oxidation reaction of the substrate L-Tyr, in presence of* $A\beta_{16}$ *10* μM *and hemin 2* μM*, vs* $[H_2O_2]$ *(left) and vs [L-Tyr] (right), in phosphate buffer 100 mM, pH 7.4 at* 25 °C.

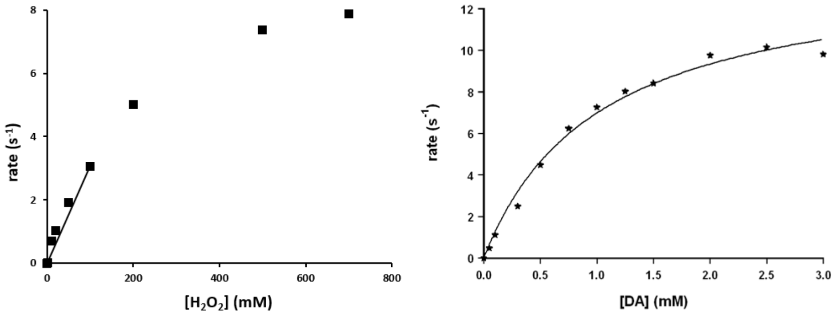

Figure S5: *trend of the initial rate of the oxidation reaction of the substrate DA, in presence of* $A\beta_{16}$ *1* μM *and hemin 0.2* μM*, vs* $[H_2O_2]$ *(left) and vs [DA] (right), in phosphate buffer 100 mM, pH 7.4 at* 25 °C.

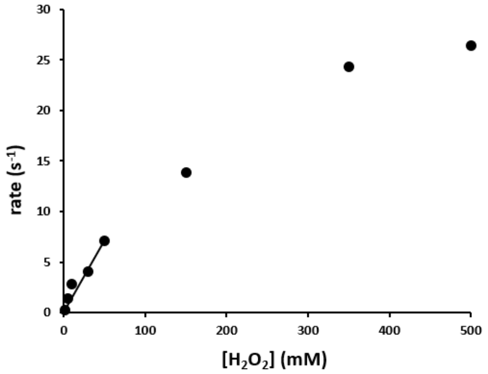

Figure S6: *trend of the initial rate of the oxidation reaction of the substrate L-DOPA, in presence of* $A\beta_{16}$ *1* μM *and hemin 0.2* μM*, vs* $[H_2O_2]$*, in phosphate buffer 100 mM, pH 7.4 at* 25 °C.

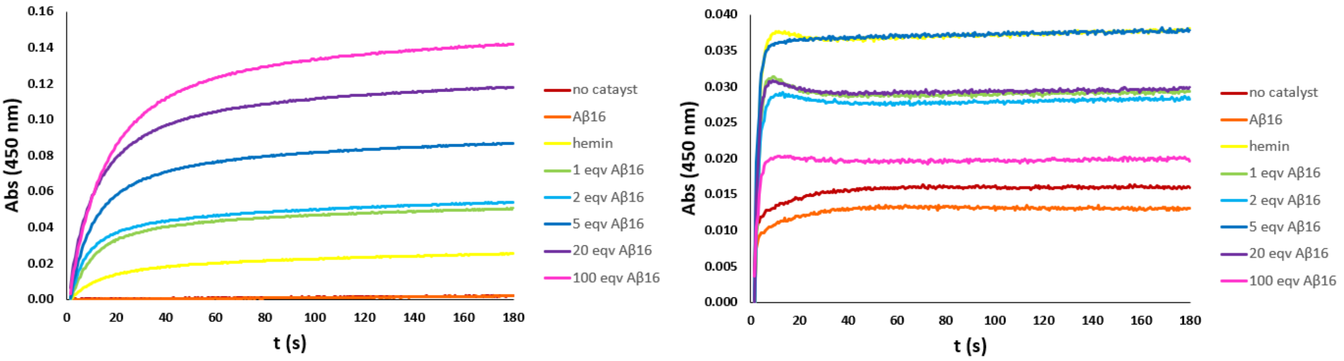

Figure S7: *kinetic trends of HPA (3 mM) nitration with H_2O_2 (50 mM) and NO_2^- (500 mM) (left) and with $ONOO^-$ (0.1 mM) (right) in phosphate buffer (100 mM, pH 7.4) at* 25 °C, *with no catalyst (brown trace) and in the presence of hemin (2 µM) (yellow trace), $A\beta_{16}$ (2 µM) (orange trace) or hemin-$A\beta_{16}$ complex with increasing amounts of $A\beta_{16}$ (1 eqv green trace, 2 eqv light blue trace, 5 eqv blue trace, 20 eqv purple trace, 100 eqv pink trace).*

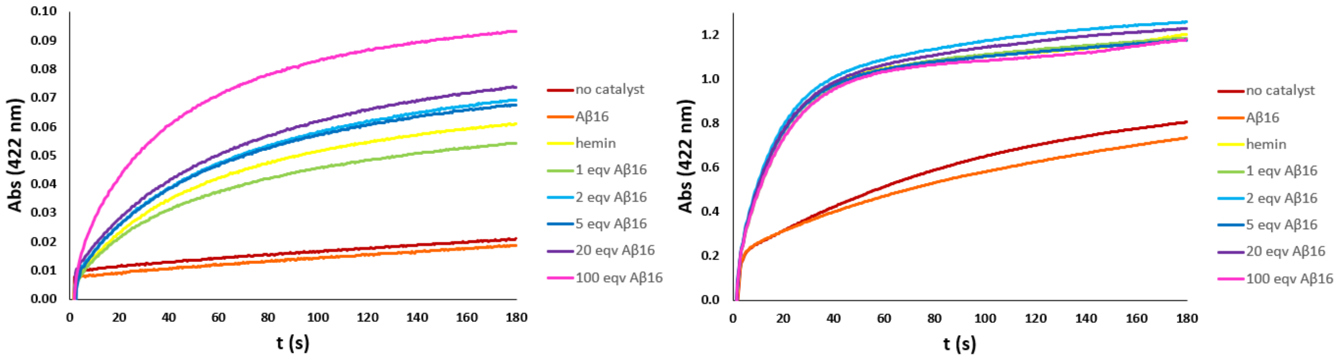

Figure S8: *kinetic trends of DA (3 mM) nitration with H_2O_2 (500 mM) and NO_2^- (500 mM) (left) and with $ONOO^-$ (1 mM) (right) in phosphate buffer (100 mM, pH 7.4) at* 25 °C, *with no catalyst (brown trace) and in the presence of hemin (0.2 µM) (yellow trace), $A\beta_{16}$ (0.2 µM) (orange trace) or hemin-$A\beta_{16}$ complex with increasing amounts of $A\beta_{16}$ (1 eqv green trace, 2 eqv light blue trace, 5 eqv blue trace, 20 eqv purple trace, 100 eqv pink trace).*

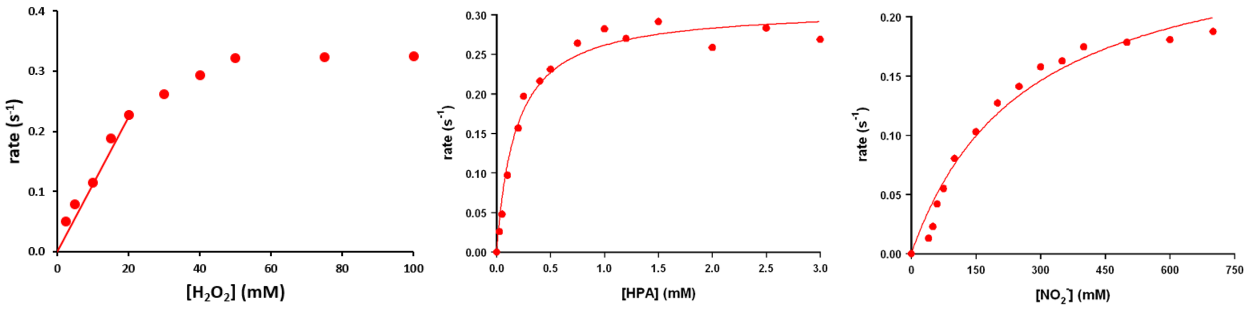

Figure S9: *trend of the initial rate of the nitration reaction with H_2O_2/NO_2^- of the substrate HPA, in presence of $A\beta_{16}$ 10 µM and hemin 2 µM, vs [H_2O_2] (left), vs [HPA] (middle) and vs [NO_2^-] (right), in phosphate buffer 100 mM, pH 7.4 at* 25 °C.

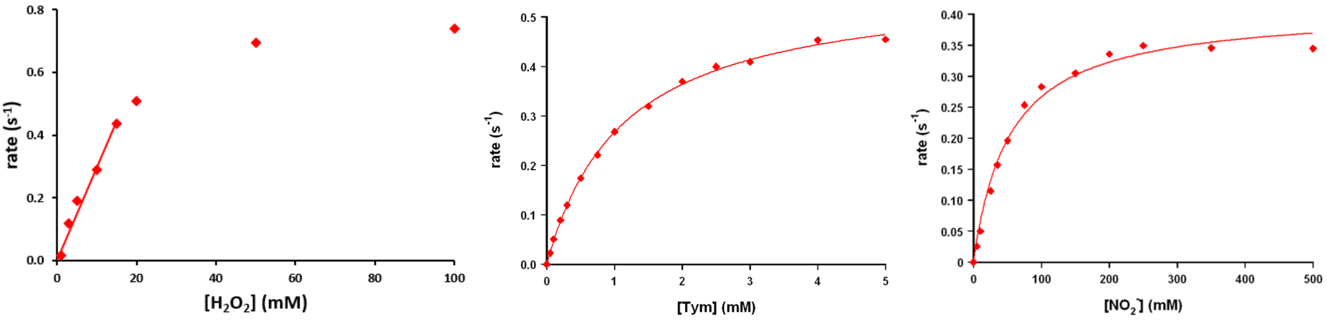

Figure S10: *trend of the initial rate of the nitration reaction with* H_2O_2/NO_2^- *of the substrate Tym, in presence of* $A\beta_{16}$ *10* μM *and hemin 2* μM*, vs* $[H_2O_2]$ *(left), vs [Tym] (middle) and vs* $[NO_2^-]$ *(right), in phosphate buffer 100 mM, pH 7.4 at* 25 °C.

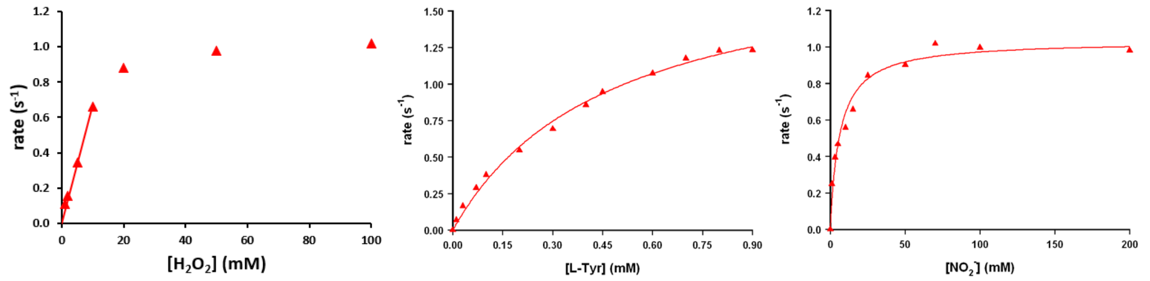

Figure S11: *trend of the initial rate of the nitration reaction with* H_2O_2/NO_2^- *of the substrate L-Tyr, in presence of* $A\beta_{16}$ *10* μM *and hemin 2* μM*, vs* $[H_2O_2]$ *(left), vs [L-Tyr] (middle) and vs* $[NO_2^-]$ *(right), in phosphate buffer 100 mM, pH 7.4 at* 25 °C.

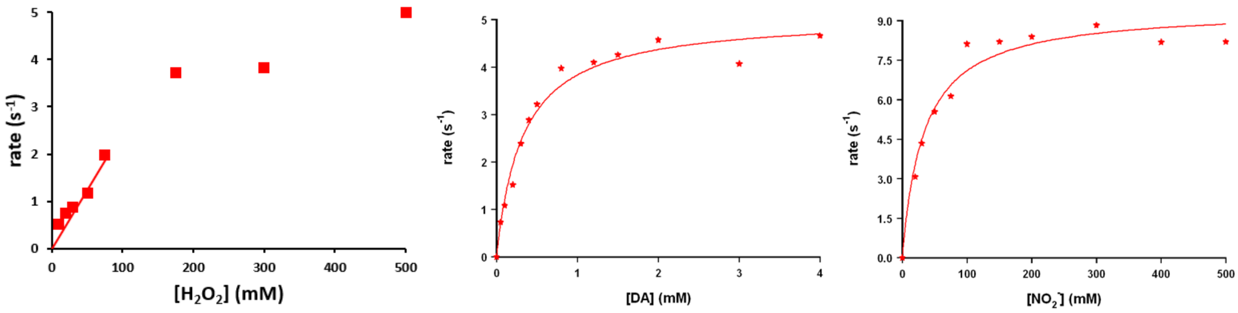

Figure S12: *trend of the initial rate of the nitration reaction with* H_2O_2/NO_2^- *of the substrate DA, in presence of* $A\beta_{16}$ *1* μM *and hemin 0.2* μM*, vs* $[H_2O_2]$ *(left), vs [DA] (middle) and vs* $[NO_2^-]$ *(right), in phosphate buffer 100 mM, pH 7.4 at* 25 °C.

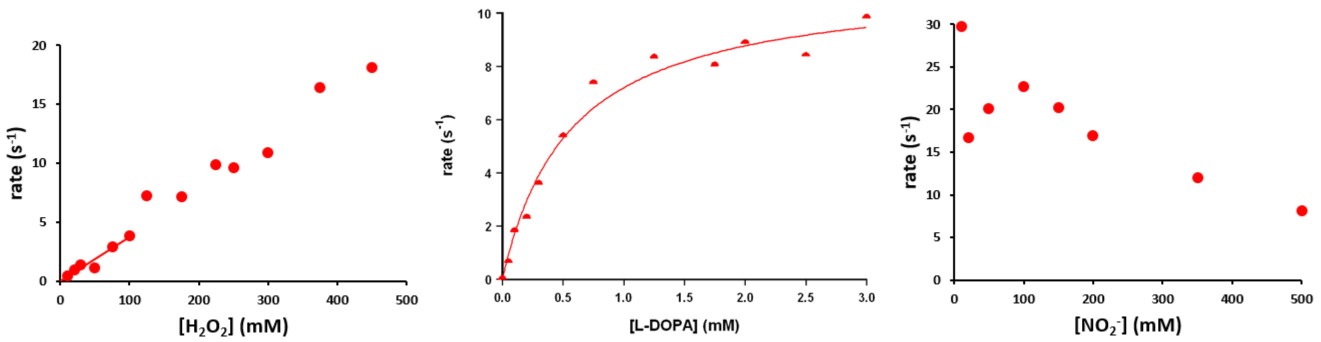

Figure S13: *trend of the initial rate of the nitration reaction with* H_2O_2/NO_2^- *of the substrate L-DOPA, in presence of* $A\beta_{16}$ *1* μM *and hemin 0.2* μM*, vs* $[H_2O_2]$ *(left), vs [L-DOPA] (middle) and vs* $[NO_2^-]$ *(right), in phosphate buffer 100 mM, pH 7.4 at* 25 °C.

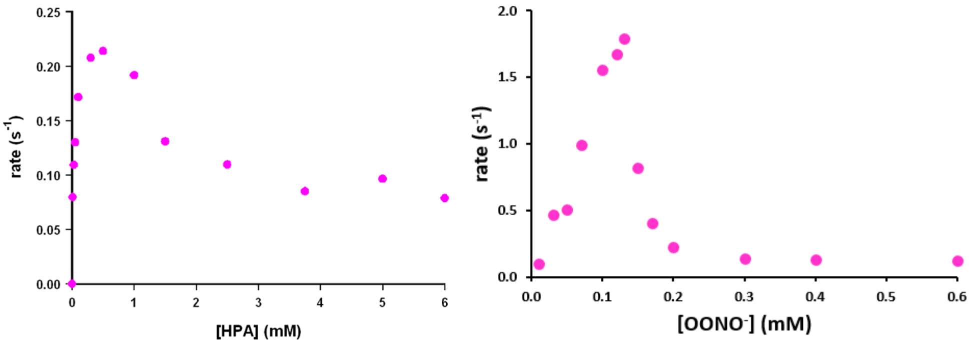

Figure S14: *trend of the initial rate of the nitration reaction with $ONOO^-$ of the substrate HPA, in presence of $A\beta_{16}$ 10 μM and hemin 2 μM, vs [HPA] (left) and vs [$ONOO^-$] (right), in phosphate buffer 100 mM, pH 7.4 at* 25 °C.

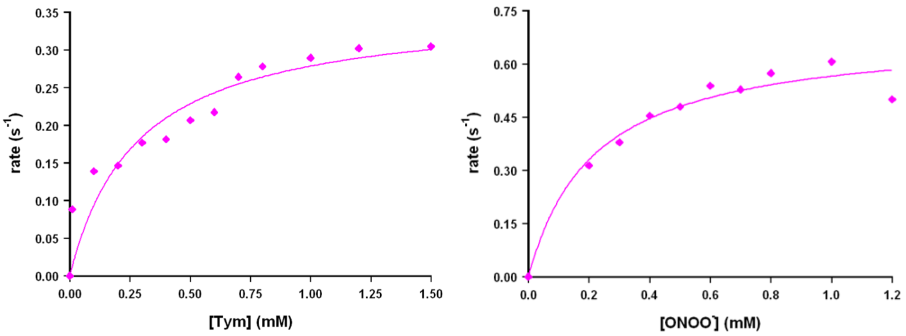

Figure S15: *trend of the initial rate of the nitration reaction with $ONOO^-$ of the substrate Tym, in presence of $A\beta_{16}$ 10 μM and hemin 2 μM, vs [Tym] (left) and vs [$ONOO^-$] (right), in phosphate buffer 100 mM, pH 7.4 at* 25 °C.

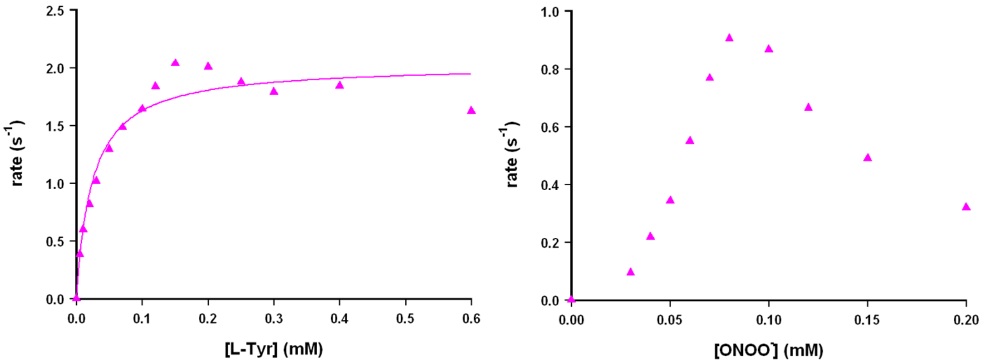

Figure S16: *trend of the initial rate of the nitration reaction with $ONOO^-$ of the substrate L-Tyr, in presence of $A\beta_{16}$ 10 μM and hemin 2 μM, vs [L-Tyr] (left) and vs [$ONOO^-$] (right), in phosphate buffer 100 mM, pH 7.4 at* 25 °C.

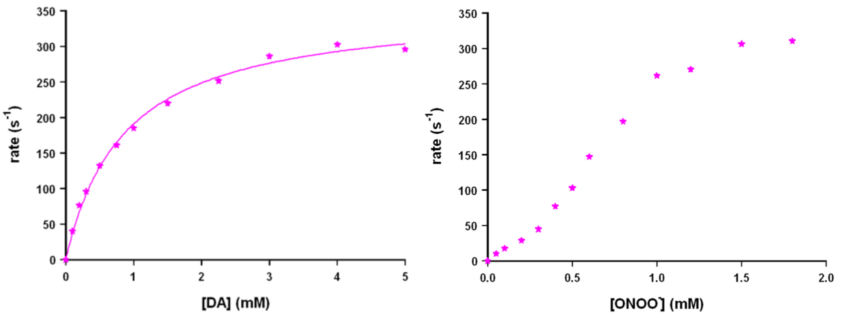

Figure S17: *trend of the initial rate of the nitration reaction with $ONOO^-$ of the substrate DA, in presence of $A\beta_{16}$ 1 μM and hemin 0.2 μM, vs [DA] (left) and vs [$ONOO^-$] (right), in phosphate buffer 100 mM, pH 7.4 at* 25 °C.

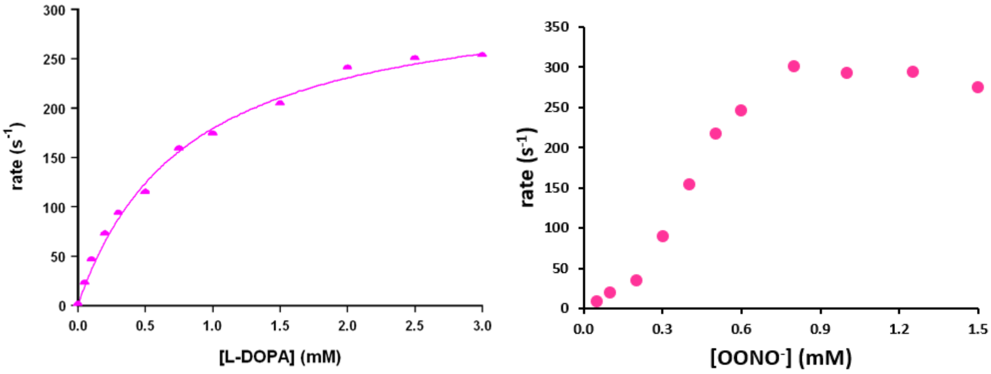

Figure S18: *trend of the initial rate of the nitration reaction with $ONOO^-$ of the substrate L-DOPA, in presence of $A\beta_{16}$ 1 μM and hemin 0.2 μM, vs [L-DOPA] (left) and vs [$ONOO^-$] (right), in phosphate buffer 100 mM, pH 7.4 at* 25 °C.

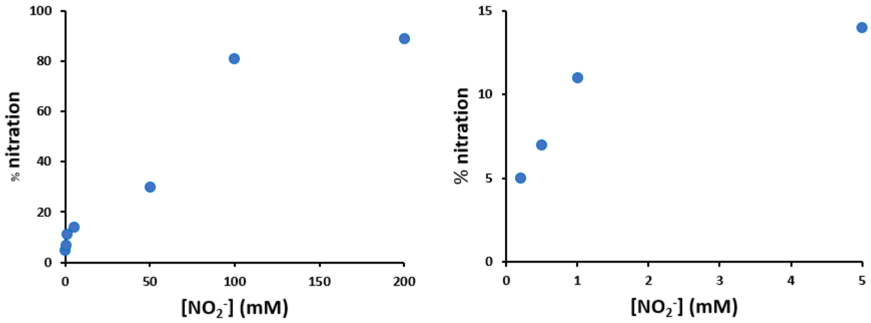

Figure S19: *variation of the nitration percentage of the peptide (10 μM), in the presence of hemin (2 μM), as a function of the concentration of nitrite and hydrogen peroxide (both varying from 0.2 to 200 mM), in phosphate buffer 100 mM, pH 7.4 after 30 min incubation at* 37 °C *(left), and magnification of the results at low concentrations (right).*

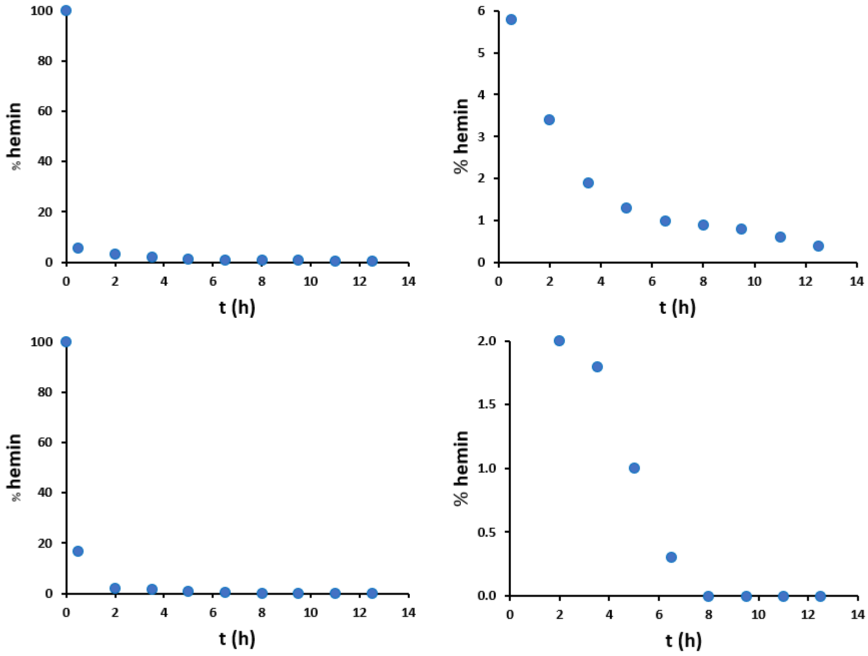

Figure S20: *percentage amount of unmodified hemin as compared to the blank sample without hydrogen peroxide, in the presence of peptide (100 μM) and hemin (20 μM), in mild (200 μM H_2O_2 / 200 μM NO_2^-) (up) and harsh (20 mM H_2O_2 / 200 mM NO_2^-) (down) conditions. On the right, magnifications of the same experimental data with reduced y-axis in the range of 0-6% hemin and 0-2% hemin for mild (up) and harsh (down) conditions, respectively, are shown.*
